# The effect of training intervention based on health belief model on self-care behaviors of women with gestational diabetes mellitus

**DOI:** 10.3389/fgwh.2024.1490754

**Published:** 2025-01-17

**Authors:** Fatemeh Mohammadkhah, Amirhossein Kamyab, Babak Pezeshki, Samira Norouzrajabi, Ali Khani Jeihooni

**Affiliations:** ^1^ Department of Community Health, Child Nursing and Aging, Ramsar School of Nursing, Babol University of Medical Sciences, Babol, Iran; ^2^ Faculty of Medicine, Fasa University of Medical Sciences, Fasa, Iran; ^3^ Department of Internal Medicine, School of Medicine, Fasa University of Medical Sciences, Fasa, Iran; ^4^ Department of Nursing, School of Nursing, Babol University of Medical Sciences, Babol, Iran; ^5^ Nutrition Research Center, Department of Public Health, School of Health, Shiraz University of Medical Sciences, Shiraz, Iran

**Keywords:** health education, health belief model, behavior, pregnant women, self-care, health-related behaviors

## Abstract

**Background:**

Gestational diabetes mellitus (GDM) is currently the most common complication of pregnancy, and the prevalence of undiagnosed hyperglycemia and overt diabetes in young women is increasing. In this regard, the present study aimed to investigate the effect of training intervention based on the health belief model of self-care behaviors in women with gestational diabetes.

**Methods:**

The present study was an interventional study, which was conducted on 160 women with gestational diabetes (80 in the interventional group and 80 in the control group), who were under treatment in healthcare centers in the city of Fasa in Fars Province, Iran, in 2022–2023. The method was simple random sampling. The collecting data tools were demographic characteristics questionnaire (age, education, occupation, monthly income of the family, gestational age (in the week), and rank of pregnancy, a knowledge assessment questionnaire, a questionnaire based on the health belief model (perceived sensitivity, perceived severity, perceived advantages, and disadvantages, self-efficiency), and the self-care behaviors questionnaire. The questionnaires were completed before the intervention and 6 weeks after the intervention. The women in the intervention group received six sessions of 50–55 min. Fasting blood sugar level and blood sugar level 2 h after the meal, A1C hemoglobin, and the need for taking insulin and the required dosage were recorded. The data were analyzed using SPSS 24, Kolmogorov-Smirnov tests (for normal distribution of data), independent *t*-test, paired *t*-test, chi-2 test, and descriptive statistics (*P* < 0.05).

**Results:**

The mean age of the participants in the intervention group and control group was 32.45 ± 4.82 and 33.16 ± 4.69, respectively. The results showed that the mean scores of all structures of the health belief model in the intervention group were significantly different from those obtained after the intervention in this group (*p* < 0.001). Also, the comparison of averages of blood sugar levels after the intervention in the two groups indicated that fasting blood sugar level, A1C hemoglobin, and blood sugar levels measured 2 h after the meal significantly decreased in the intervention group (*p* < 0.001). The need to increase the dosage of insulin in the intervention group was lower than in the control group.

**Conclusions:**

according to the results, the health belief model was effective in improving clinical results of self-care behaviors in women with gestational diabetes. HBM played an important role in understanding what care and support the women need. Therefore, the incidence of various diseases can be prevented and mothers with GDM can experience such vulnerability less than before. It can also be used as a model to design, implement, and monitor health programs for women with gestational diabetes.

## Background

Gestational Diabetes Mellitus (GDM) is currently the most common medical complication in pregnancy, and the prevalence of unrecognized hyperglycemia and even overt diabetes is on the rise in young women (1). Diagnosis is usually made using an oral glucose tolerance test (OGTT). Correcting diet and increasing physical activities are the primary risk factors (2). The risk factors of GDM include overweight and obesity (BMI ≥ 25 kg/m^2^), old age of the mother, history of any type of diabetes in the family, blood pressure caused by pregnancy, history of stillbirth, polycystic ovary syndrome, history of abortion, age over 25 years old, and history of premature birth (3). GDM affects about 16.5% of pregnancies in the world, which increases with the increasing obesity epidemic (4). The standardized global prevalence of GDM was 14%. The standardized prevalence of GDM in low, middle, and high-income countries was 12.7%, 9.2%, and 14.2%, respectively (5).

The prevalence of diabetes in Iran was 7.9%. The highest prevalence was reported in Tehran (23.99%), followed by Mazandaran province (23.13%), while the lowest prevalence was observed in Ardabil province (1.33%) (6). Lifestyle changes are essential in the management of GDM. The present study sought to present a general perspective of lifestyle changes, which can be suggested for GDM management. The suggested diet for women with GDM should contain adequate macronutrients and micronutrients to support fetal growth while limiting postprandial glucose and encouraging appropriate maternal weight gain (7). However, there is a distinct knowledge-behavior gap among women with gestational diabetes, while they showed a lack of knowledge about the required corrective changes in their lifestyle (8).

One effective measure to promote self-care behaviors in GDM is training interventions (9). The first step in designing a training program is selecting an educational one. Self-efficiency, perceived barriers, perceived sensitivity, and perceived severity are the most important predicting factors in health behaviors. The factors are the constructs of the health belief model (HBM) in health education, which are used to examine the reasons for accepting or rejecting health issues from the public (10). The previous studies have indicated the effectiveness of training interventions of GDM self-management in improving knowledge, attitude, and performance of pregnant women, weight loss after being pregnant, and attendance in monitoring programs of OGTT to 12 weeks after delivery. Moreover, a significant improvement was found in the constructs of the health belief model after the intervention (11–14).

Healthcare staff can use the health belief model to increase pregnant women's understanding of the risk of GDM. This enables them to use strategies to make changes in a healthy lifestyle. Understanding the interactions of health belief model elements contributes to understanding the risks for women with GDM. Therefore, women with GDM should adopt health-behavioral strategies to prevent or slow down GDM. HBM plays an important role in understanding what types of support and care these women need. Therefore, the incidence of several diseases can be prevented, and the experiences of mothers with GDM can be recovered during this vulnerability (15). Because GDM is common in pregnant women in Iran and education and HBM-based interventions have been shown to improve self-care behaviors, the goal of this study was to look into how HBM-based training interventions affect self-care behaviors in women with GDM.

## Methods

The present quasi-experimental study was conducted on 160 women with GDM who were referred to healthcare centers in the city of Fasa in Fars Province, Iran, in 2022–2023. For sampling, two centers (out of 6) were randomly selected (one for the intervention group and one for the control group). The sampling was based on the household record number of women using a random sampling method. The samples were invited to gather in the centers on a determined day. Then, they were explained the goals of the study, and the written consent forms were obtained. The sample size, according to a study by Dousti et al. (14), and using the formula of variables mean comparison and considering the samples fall, was determined to be 80 in each group. The inclusion criteria were: being diabetic between 26 and 30 weeks gestation, willingness to participate in the study, living in Fasa, absence of any history of premature birth, the threat of abortion, multiple births, and intrauterine growth retardation in the last pregnancy, and absence of chronic diseases before and during pregnancy (such as known cardiopulmonary disease, seizures, thrombophilia, pulmonary embolism, chronic blood pressure, overt diabetes, hemoglobin less than 100 mg/L, and incompetent cervix). The exclusion criteria were: being restricted or prohibited from performing self-care behaviors; unwillingness to continue participating in the study; absenteeism for more than one session from training sessions; immigration; and lack of access.

**Figure 1:**
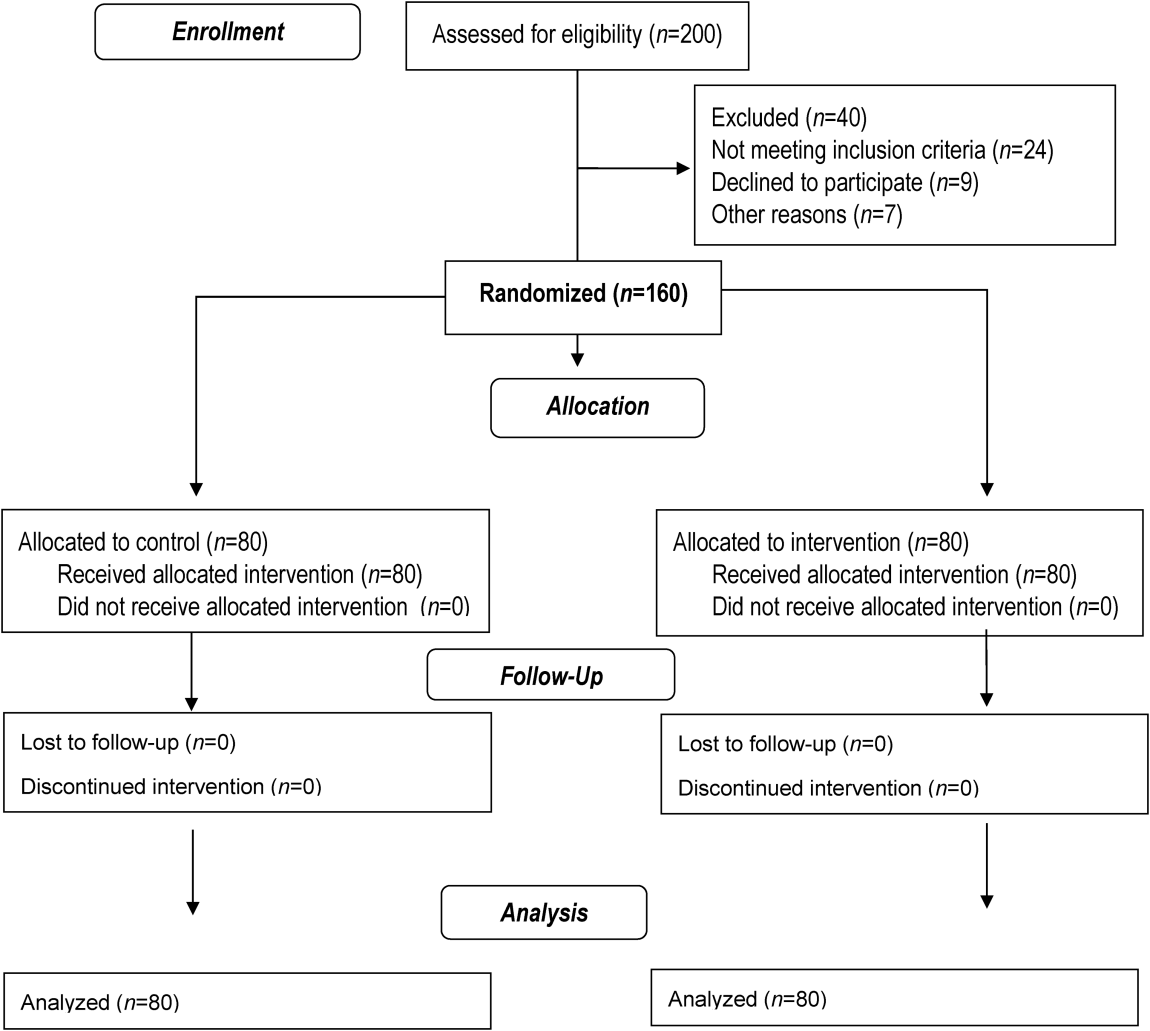
Flow chart of study.

According to the inclusion criteria, 160 people were finally included in the study, which were divided into two control and intervention groups (80 people in each group). Then the intervention group received the necessary training, and at the end of the study, the results of the two groups were compared with the relevant statistical tests analyzed (Figure 1).

### Data collection tools

The collecting data tools included demographic characteristics [age, education, occupation, household monthly income, gestation age (week), parity], a knowledge assessment questionnaire, an HBM-based questionnaire, and a self-care behaviors questionnaire with confirmed validity and reliability (16–20).

The knowledge assessment questionnaire is composed of 15 items with three options (yes, no, no idea). Two points were allocated to “yes” answers and none to “no” and “no idea” answers. The higher score indicated the mothers' high level of knowledge about the self-care behaviors of GDM. The HMB-based model questionnaire included: perceived sensitivity (8 items), perceived severity (8 items), perceived benefits (10 items), perceived barriers (8 items), perceived self-efficiency (12 items), and act directions (7 items). The items were scored using a 5-point Likert scale (from strongly disagree = 1 to strongly agree = 5). The self-care behaviors of the GDM questionnaire (healthy diet, physical activity, monitoring blood pressure, injecting insulin, and correct consumption of oral medications, etc.) included 15 items with yes and no options. The scores were between 0 and 15. Selecting the intervention and control groups, the goals of the study, and the method were explained to the pregnant women and the healthcare staff of the centers. The two groups completed the questionnaires. In the present study, the intervention group received individual and group counseling on HBM-based self-care behaviors in addition to routine care. The counseling sessions were held three times a week (each session 50–55 min) for six sessions. The counseling content was related to the people's activities in having a healthy diet, physical activity, monitoring blood pressure, injecting insulin, correct consumption of oral medications, and lack of smoking.

### Training program

The training program for the intervention group was designed as a structured and theory-driven initiative to improve self-care behaviors in women with GDM using the HBM. The intervention consisted of six sessions, each lasting 50–55 min, conducted three times a week over a two-week period. A multidisciplinary team facilitated the sessions, including a Ph.D. expert in health education and promotion, a specialist in adult endocrinology, a nutritionist, and a gynecologist. The training program also incorporated participation from family members and healthcare staff to foster a supportive learning environment. The content of the sessions was comprehensive and tailored to address key aspects of self-care for women with GDM. The program began with an introduction to GDM, highlighting its causes, potential complications for the mother and fetus, and the importance of effective blood glucose management during pregnancy. This foundational knowledge aimed to help participants understand their condition and the critical need for self-care. Dietary management was a significant focus of the program, with participants learning about selecting low-glycemic-index foods, planning balanced meals with appropriate portions of macronutrients, and incorporating essential vitamins and minerals. Practical strategies for managing cravings and avoiding high-sugar snacks were also emphasized to ensure adherence to a GDM-friendly diet.

Physical activity was another critical component of the training. Participants were guided on incorporating moderate, safe exercises into their daily routines, such as walking or prenatal yoga, to help regulate blood sugar levels. They also received detailed instruction on monitoring their blood glucose, including the correct use of glucometers for fasting and postprandial measurements. These sessions aimed to improve participants' ability to understand glucose readings, recognize abnormal patterns, and appreciate the importance of consistent monitoring to reduce complications. Medication and insulin management were addressed extensively in the program. Participants were taught proper techniques for administering insulin injections, adhering to prescribed oral medications, and overcoming fears or misconceptions about insulin therapy. Additionally, the training highlighted the harmful effects of smoking during pregnancy, especially for those with GDM, and encouraged participants to adopt broader lifestyle modifications, including stress management practices.

The training sessions employed interactive methods to engage participants and reinforce learning. Group discussions, role-playing, and Q&A sessions allowed participants to share experiences, clarify doubts, and strengthen their understanding. Practical demonstrations, such as using glucometers and planning meals, were included to enhance confidence and skill development. In one session, husbands, healthcare staff, and the participants' diabetes specialists were invited to participate, creating a collaborative and inclusive environment.

To support the learning process, participants were provided with a training booklet and a CD containing session summaries, practical tips for daily self-care, instructions on using medical devices, and GDM-friendly recipes. These materials served as a reference for participants to reinforce their knowledge and practice what they had learned. Participants were also encouraged to document their daily self-care activities, including blood glucose readings, meal plans, physical activities, and insulin dosages, using a structured form. This practice not only promoted accountability but also allowed facilitators to provide personalized feedback and guidance.

The training program included a six-week follow-up period to ensure continuity and reinforce behavioral changes. Weekly telephone calls and in-person reminders were conducted to address any challenges, answer questions, and provide ongoing motivation. Through this multifaceted approach, the training program equipped participants with the knowledge, skills, and support necessary to manage their GDM effectively, leading to sustainable improvements in self-care behaviors and clinical outcomes.

### Data analysis

The data were analyzed using SPSS24, Kolmogorov-Smirnov test (for normal distribution of data), independent *t*-test, paired *t*-test, Chi-2 test, and descriptive statistics.

## Results

160 pregnant women (80 in the intervention group and 80 in the control group) participated in this study. The mean age of the women in the intervention group was 32.45 ± 4.82, and 33.16 ± 4.69 in the control group. The independent *t*-test showed no significant difference between the two groups (*p* = 0.304). The mean gestation age (week) in the intervention group was 28.4 ± 1.7 and 27.9 ± 2.1 in the control group, indicating no significant difference (*p* = 0.516).

Of the 160 participants included in the study, 115 (71.9%) were managed with dietary interventions alone, while 45 (28.1%) required insulin therapy in addition to dietary management. The proportion of participants requiring insulin therapy was similar between the intervention group (22 patients, 27.5%) and the control group (23 patients, 28.8%), with no significant difference (*p* = 0.678).

The results of the chi-2 square showed no significant difference between the two groups in terms of education, occupation, household monthly income, and pregnancy rank (Table 1).

**Table 1:** The comparison of demographic characteristics of the women under study in the two groups.

Variable		Intervention group		Control group		*p*-value
		*n* = 80		*n* = 80		
		No.	Percentage	No.	Percentage	
Occupation	Housewife	64	80	68	85	0.274
	Employed	16	20	12	15	
Monthly income	Less than 40 million rials	22	27.5	20	25	0.245
	40–80 million rials	36	45	35	43.75	
	More than 80 million rials	22	27.5	25	31.25	
Education	Illiterate	1	1.25	2	2.5	0.192
	Elementary school	14	17.5	8	10	
	Guidance school	18	22.5	16	20	
	High school	31	38.75	42	52.5	
	Academic	16	20	12	15	
Pregnancy rank	First	26	32.5	28	35	0.186
	Second	42	52.5	38	47.5	
	Third and higher	12	15	14	17.5	

The comparison of average blood glucose indicators after the intervention in the two groups showed that the average fasting blood glucose, A1C hemoglobin, and blood glucose 2 h after the meal significantly decreased in the intervention group. The consumed insulin dose before and after the intervention showed no significant difference in the two groups; however, the increase in insulin consumption in the intervention group was lower than in the control group (Table 2).

**Table 2:** The comparison of average blood glucose indicators and insulin dose in the two intervention and control groups (before and after the intervention).

Variable	Group	Before the intervention	After the intervention	*p*-value
		M ± SD	M ± SD	
Blood glucose (mg/dl)	Intervention	89.40 ± 14.92	84.21 ± 12.09	0.001
	Control	88.98 ± 15.30	88.02 ± 15.40	0.239
	*p*-value	0.201	0.001	
Hemoglobin percentage	Intervention	5.4 ± 0.7	5.51 ± 0.4	0.001
	Control	5.5 ± 0.7	5.7 ± 0.6	0.001
	*p*-value	0.07	0.001	
2 h blood glucose (mg/dl)	Intervention	131.6 ± 21.3	118.7 ± 16.1	0.001
	Control	131.4 ± 22.2	137.1 ± 20.90	0.001
	*p*-value	0.368	0.001	
Insulin dose (unit per day)	Intervention	8.3 ± 5.8	13.2 ± 7.7	0.001
	Control	8.7 ± 5.2	21.4 ± 12.1	0.001
	*p*-value	0.168	0.001	

The results showed no significant difference between the two groups before the intervention in knowledge, perceived sensitivity, perceived severity, perceived benefits, perceived barriers, perceived self-efficiency, art direction, and self-care behaviors of GDM. But, after the intervention, the intervention group showed a significant increase in all variables except for the perceived barriers (Table 3).

**Table 3:** The comparison of the mean scores of awareness, HBM, and self-care behaviors model constructs in the two groups before and after the intervention.

Variable	Group	Before the intervention	After the intervention	*p*-value
		M ± SD	M ± SD	
Knowledge	Intervention	12.44 ± 2.18	24.72 ± 2.32	0.001
	Control	14.12 ± 2.08	15 ± 12.10	0.312
	*p*-value	0.206	0.001	
Perceived sensitivity	Intervention	17.22 ± 3.12	34.20 ± 3.58	0.001
	Control	16.65 ± 3.27	17.24 ± 3.31	0.244
	*p*-value	0.24	0.001	
Perceived sensitivity	Intervention	15.55 ± 3.10	35.16 ± 3.43	0.001
	Control	17.62 ± 3.27	18.60 ± 3.30	0.220
	*p*-value	0.216	0.001	
Perceived benefits	Intervention	19.43 ± 3.69	42.48 ± 3.72	0.001
	Control	20.45 ± 3.71	23.80 ± 3.77	0.174
	*p*-value	0.218	0.001	
Perceived barriers	Intervention	28.12 ± 3.03	10.17 ± 3.14	0.001
	Control	29.33 ± 3.01	27.56 ± 3.06	0.217
	*p*-value	0.212	0.001	
Perceived self-efficiency	Intervention	26.45 ± 4.59	50.78 ± 4.62	0.001
	Control	23.88 ± 4.56	24.90 ± 4.60	0.213
	*p*-value	0.170	0.001	
Act directions	Intervention	14.56 ± 2.18	29.60 ± 2.25	0.001
	Control	15.02 ± 2.22	13.40 ± 2.24	0.227
	*p*-value	0.218	0.001	
Self-care behaviors	Intervention	6.80 ± 1.13	13.18 ± 1.23	0.001
	Control	6.68 ± 1.16	6.81 ± 1.20	0.422
	*p*-value	0.478	0.001	

## Discussion

The present study aimed to investigate the effect of training interventions based on the health belief model on self-care behaviors in women with gestational diabetes mellitus. This study's results show that the interventional program worked to improve all aspects of the HBM. These results are similar to those of Mohebbi et al. (2019) (21), Nkomani et al. (2021) (12), and Desoky et al. (2022) (20). In 2019, Mohebbi et al. found that an HBM-based training intervention made all the parts of the model, self-management, and the clinical results of HbA1c much better in the intervention group (21). Nkomani et al. (2021) demonstrated the effectiveness of diabetes self-management training interventions on the knowledge, attitude, and performance of pregnant women referred to public healthcare centers (12).

However, Desoky et al.'s study from 2022 showed a strong link between the scores on knowledge, lifestyle, and HBM constructs before and after using the GDM training package based on HBM (20). In this study, a unique, individual, and academic training program based on a model-based training package was performed for the intervention group in six 50–55 min sessions. The participants gained an understanding of the significance of attending training sessions and blood glucose tests, which subsequently influenced their self-care behaviors and led to improvements in their clinical outcomes. The comparison of mean scores of knowledge before and after the intervention showed a significant difference between the two groups, which was in line with the results of a study by Khiyali et al. (2017) (22), Desoky et al. (2022) (20), and Smitha et al. (2015) (23). In other words, the results of the present study showed that training the GDM self-care behaviors could affect people's knowledge about self-care. In this study, the training sessions focused on improving people's knowledge about maintaining a healthy diet, engaging in physical activity, monitoring blood glucose, injecting insulin, correctly consuming oral medication, and refraining from smoking. Khiyali et al. (2017) showed that training interventions based on the preventive behaviors of GDM increased the knowledge of the participants in the intervention group (22). In the study of Desoky et al. (2022), the HBM-based training packages improved knowledge in women with GDM (20). The study of Smitha et al. (2015) also improved knowledge in the target group (23). The comparThere was a big difference between the intervention and control groups in terms of mean scores for perceived sensitivity before and after the intervention. This was in line with what other studies by Helm et al. (2022) (24), Mohebbi et al. (2019) (21), and Tawfik et al. (2017) (25) found. In other words, the present study's results indicate that GDM self-care behaviors can influence perceived sensitivity, potentially contributing to the development of problematic bPeople may understand the seriousness of their health situations and feel vulnerable, so trainers should assist them in facing reality.

In a study by Khiyali et al. (2017), the training intervention of improving GDM preventive behaviors can improve perceived severity in the target group (22). Also, Desoky et al. (2022) showed that an HBM-based training package for improving lifestyle in women with GDM improves the construct of perceived severity in the intervention group (20). One more study, by Mohebbi et al. (2019), found that self-management training based on HBM made the concept of perceived severity better in a group of women with GDM (21). The comparison of mean scores of perceived threat (perceived benefits and barriers) before and after the intervention showed that there was a significant difference between the two groups. This result was consistent with the results of studies by Matsuzaki et al. (2018) (26), Desoky et al. (2022) (20), and Mohebbi et al. (2019) (21). They indicated the effectiveness of training in improving perceived threats (perceived benefits and barriers) in the intervention group. Perceived benefits are defined as a person's belief in the efficiency of the recommended measures in reducing threats and the seriousness of the issue (27). After describing the role of self-care behaviors, it appears that the score of perceived benefits significantly increased, leading to a reduction in the problems faced by women with GDM in the intervention group. Also, perceived barriers were one of the strongest and most important predictors of behavior in students, which should receive consideration in designing training programs (28). According to the results of the present study, perceived barriers showed a significant reduction in the intervention group compared to the control group. Therefore, we can use this model effectively to reduce perceived barriers to GDM self-care behaviors. Matsuzaki et al. (2018) investigated the effects of a yoga and nutrition training program on the consequences of pregnancy among women with GDM and indicated the effectiveness of training in improving perceived threats (benefits and barriers) in the intervention group (26).

We found a big difference between the intervention and control groups when we looked at the mean scores of perceived self-efficiency before and after the intervention. This was in line with what other studies (29). Self-efficiency is defined as people's confidence in their abilities in following a behavior (30). People's belief in performing correct GDM self-care behaviors can effectively enhance their self-efficiency. The self-efficiency construct can serve as the foundation for behavior development, as it has a strong correlation with the expression of behavior. An HBM-based training program may help improve self-efficiency and adopt self-care behaviors in the target group. As indicated in this study, the people recorded their self-care behaviors in specific forms, and the follow-up process lasted for 6 weeks via telephone and in-person reminders, leading to improved self-efficiency in the intervention group.

Skar et al. (2018) investigated pregnant women's experiences of using a smartphone application (pregnancy application) to manage GDM and indicated the effectiveness of the program in improving the construct of perceived self-efficiency in the target group (31). Desoky et al. (2022) showed that the HBM-based training package used to improve lifestyle in women with GDM improved the construct of perceived self-efficiency in the intervention group (20).

The comparison of the means scores of act direction before and after the intervention showed a significant difference between the two groups, which was in line with the results of studies by Adb-Elhakam et al. (2022) (32), Mohebbi et al. (2019) (21), and Desoky et al. (2022) (20), indicating the effectiveness of training intervention in improving the mean scores of act directions in the intervention group. A Ph.D. expert in health education and promotion, a specialist in adult endocrinology, a nutritionist, and a gynecologist implemented the training program. In one of the training sessions, the husbands, healthcare staff of the centers, and the diabetes doctor were also present. The follow-up process, which lasted for 6 weeks, was performed via telephone and in-person reminder., which seems to have improved the construct of act directions in this study. Abd-Elhakam et al. (2022) showed the effects of an HBM-based interventional program on preventing premature birth in pregnant women, indicating the effectiveness of the intervention in improving the mean scores of act directions in the intervention group (32). In the study of Desoky et al. (2022), the HBM-based training package used to improve lifestyle in women with GDM improved the mean scores of act directions in the intervention group (20). In the study of Mohebbi et al. (2019), the HBM-based self-management training intervention program conducted for women with GDM improved the mean scores of act directions in the intervention group (21).

The comparison of the mean scores of self-care behaviors before and after the intervention showed a significant difference, which was in line with the results of studies by Skar et al. (2018) (31), Mohebbi et al. (2019) (21), and Matsuzaki et al. (2018) (26), indicating the effectiveness of the training intervention in improving the mean scores of self-care behaviors in the intervention group. Therefore, we recommend implementing appropriate training interventions, utilizing behavioral models and theories like the health belief model, to enhance self-care performance in women with GDM and enhance clinical outcomes like blood glucose indicators and insulin dosage in the group. Matsuzaki et al. (2018) investigated the effects of a yoga training program and nutrition on the consequences of pregnancy in pregnant women and indicated the effectiveness of mean scores of self-care behaviors in the intervention group (26). Skar et al. (2018) investigated pregnant women's experiences of using a smartphone program (the pregnancy application) to manage GDM. The results of the study indicated the effectiveness of training in the improvement of the mean scores of self-care behaviors in the target group (31). Mohebbi et al. (2019) showed that the HBM-based self-management training intervention program improved the mean scores of self-care behaviors in women with GDM in the intervention group (21).

This model enables us to propose a predictive theoretical model of health behavior, which we can use as a foundation to optimize the interventions performed on women with GDM. The self-reported nature of health behaviors was a limitation of the study. Therefore, we suggest conducting the same study in other populations. One of the strengths of the present study was studying the vulnerable groups of problem-based pregnant women and applying the theory-based model of HBM to improving self-care behaviors in women with GDM.

## Conclusion

The results indicate that HBM-based health education significantly increased the mean scores of the HBM-based model's constructs. Therefore, health and medical authorities need to adopt appropriate theory-based training programs to create and improve the self-care behaviors of this target group. Health experts, physicians, and other healthcare staff, as well as the media, as an important source to communicate health messages to the public, can play a more effective role in improving the health of women with GDM by providing health-related information. In this regard, considering theory-based educational content of self-care behaviors for women with GDM in the educational content for pregnant women and executive guidelines and protocols of healthcare centers can be helpful and effective.

## Data Availability

Data is available from the corresponding author, upon reasonable request.

## References

[1] McIntyre HD Catalano P Zhang C Desoye G Mathiesen ER Damm P. Gestational diabetes mellitus. Nat Rev Dis Primers. (2019) 5(1):47. 10.1038/s41572-019-0098-8 31296866

[2] Shepherd E Gomersall JC Tieu J Han S Crowther CA Middleton P. Combined diet and exercise interventions for preventing gestational diabetes mellitus. Cochrane Database Syst Rev. (2017) 19(11). 10.1002/14651858.CD010443.pub3 PMC6485974 29129039

[3] Choudhury AA Rajeswari VD. Gestational diabetes mellitus-A metabolic and reproductive disorder. Biomed Pharmacother. (2021) 143:112183. 10.1016/j.biopha.2021.112183 34560536

[4] Choi MJ Yu J Choi J. Maternal pre-pregnancy obesity and gestational diabetes mellitus increase the risk of childhood obesity. Children. (2022) 9(7):928. 10.3390/children9070928 35883912 PMC9323254

[5] Wang H Li N Chivese T Werfalli M Sun H Yuen L IDF diabetes atlas: estimation of global and regional gestational diabetes mellitus prevalence for 2021 by international association of diabetes in pregnancy study group’s criteria. Diabetes Res Clin Pract. (2022) 183:109050. 10.1016/j.diabres.2021.109050 34883186

[6] Mohaddeseh D Mohammadreza F Mozhgan R Mahin B Abdolghani A. Prevalence of gestational diabetes Mellitus in Iran: a systematic review and meta-analysis study. J Diabetic Nurs. (2022) 10(2).

[7] Rasmussen L Poulsen CW Kampmann U Smedegaard SB Ovesen PG Fuglsang J. Diet and healthy lifestyle in the management of gestational diabetes mellitus. Nutrients. (2020) 12(10):3050. 10.3390/nu12103050 33036170 PMC7599681

[8] Rhoads-Baeza ME. Assessing the Knowledge, Attitudes, and Behaviors of Pregnant Hispanic Women: Developing an Effective Educational Intervention for Gestational Diabetes. Berlin, Germany: University of Illinois at Urbana-Champaign (2008).

[9] Mirghafourvand M Zandinava H Shafaei FS Mohammad-Alizadeh-Charandabi S Ghanbari-Homayi S. Effectiveness of self-care training on pregnancy consequences in gestational diabetes: a randomized controlled clinical trial. Shiraz E-Med J. (2019) 20(6):e82704. 10.5812/semj.82704

[10] Anuar H Shah S Gafor H Mahmood M Ghazi HF. Usage of Health Belief Model (HBM) in health behavior: a systematic review. Malays J Med Health Sci. (2020) 16(11):2636–9346.

[11] Mahmoud NM Mohammed Y Essa R. The relationship between health belief model and compliance with therapeutic regimen among diabetic pregnant women. Int J Res Health Sci Nurs. (2018) 4(2):40–63.

[12] Nkomani S Ruskaniko S Blaauw R. The impact of existing diabetes self-management education interventions on knowledge, attitudes and practices in public health care institutions in Harare, Zimbabwe. South Afr J Clin Nutr. (2021) 34(1):27–33. 10.1080/16070658.2019.1641272

[13] Carolan-Olah M Sayakhot P. A randomized controlled trial of a web-based education intervention for women with gestational diabetes mellitus. Midwifery. (2019) 68:39–47. 10.1016/j.midw.2018.08.019 30343264

[14] Dousti F Maleki A Chiti H Faghihzadeh S Taheri SS. Investigation of the effect of individual counseling of physical activity based on theory of planned behavior on glycemic indexes in women with gestational diabetes: a randomized clinical trial. Qom Univ Med Sci J. (2018) 12(9):26–37. 10.29252/qums.12.9.26

[15] Craig L Sims R Glasziou P Thomas R. Women’s experiences of a diagnosis of gestational diabetes mellitus: a systematic review. BMC Pregnancy Childbirth. (2020) 20:1–15. 10.1186/s12884-020-2745-1 PMC7006162 32028931

[16] Zeinali A Dolatian M Janatiataie P Shams J Nasiri M. Comparison of health-promoting lifestyle and irrational health beliefs in healthy pregnant women and gestational diabetes mellitus. J Educ Health Promot. (2021) 10(1):262. 10.4103/jehp.jehp_1565_20 34485559 PMC8395982

[17] Kim Y Lee JL Jang IS Park S. Knowledge and health beliefs of gestational diabetes mellitus associated with breastfeeding intention among pregnant women in Bangladesh. Asian Nurs Res (Korean Soc Nurs Sci). (2020) 14(3):144–9. 10.1016/j.anr.2020.06.001 32645378

[18] Kordi M Banaei Heravan M Asgharipour N Mazloum SR Akhlaghi F. Relationship between self-care behaviors and coping styles in women with gestational diabetes. J Mazandaran Univ Med Sci. (2016) 26(139):190–202.

[19] Karbalai Harafteh F Karami Mohajeri Z Kia S. The effect of self-care training on perceived stress, health literacy, and self-care behaviors in women with gestational diabetes. Community Health J. (2020) 14(2):30–9. 10.22123/chj.2020.221529.1450

[20] Mostafa Abdelmonem Desoky M Morsy Salim Metwally H Abdo Hussien A. Effect of health belief model based educational package on lifestyle among gestational diabetic women. Egypt J Health Care. (2022) 13(4):1277–92. 10.21608/ejhc.2022.269396

[21] Mohebbi B Tol A Sadeghi R Mohtarami SF Shamshiri A. Self-management intervention program based on the Health Belief Model (HBM) among women with gestational diabetes mellitus: a quazi-experimental study. Arch Iran Med. (2019) 22(4):168–73. 31126174

[22] Khiyali Z Manoochri M Khani A Babaei Heydarabadi A Mobasheri F. Educational intervention on preventive behaviors on gestational diabetes in pregnant women: application of health belief model. Int J Pediatr. (2017) 5(5):4821–31. 10.22038/ijp.2016.7750

[23] Smitha K D’Almeida S. Effectiveness of self-instructional module on knowledge regarding self-care management of gestational diabetes mellitus among antenatal women visiting selected antenatal clinic at mangalore”. Int J Adv Nurs Manage. (2015) 3(1):42–5.

[24] Helm MM Izuora K Basu A. Nutrition-education-based interventions in gestational diabetes: a scoping review of clinical trials. Int J Environ Res Public Health. (2022) 19(19):12926. 10.3390/ijerph191912926 36232232 PMC9564999

[25] Tawfik MY. The impact of health education intervention for prevention and early detection of type 2 diabetes in women with gestational diabetes. J Community Health. (2017) 42:500–10. 10.1007/s10900-016-0282-7 27743337

[26] Matsuzaki M Kusaka M Sugimoto T Shiraishi M Kobayashi R Watanabe S The effects of a yoga exercise and nutritional guidance program on pregnancy outcomes among healthy pregnant Japanese women: a study protocol for a randomized controlled trial. J Altern Complementary Med. (2018) 24(6):603–10. 10.1089/acm.2017.0119 29443533

[27] Sheeran P Harris PR Epton T. Does heightening risk appraisals change people’s intentions and behavior? A meta-analysis of experimental studies. Psychol Bull. (2014) 140(2):511. 10.1037/a0033065 23731175

[28] Newsome AN Gilliard T Phillips A Dedrick R. Understanding the perceptions of sedentary college students’ engagement in physical activity: application of the theory of planned behavior. J Am Coll Health. (2023) 71(9):2813–22. 10.1080/07448481.2021.1998069 34788584

[29] Rahimzadeh A Faghih Solaimani P Rahmani K Bagheri S. Effect of a training intervention program designed based on health belief model on adopting behaviors preventing dental caries in students. Iran J Health Educ Health Promot. (2018) 6(3):266–76. 10.30699/acadpub.ijhehp.6.3.266

[30] Kudusheva N Amanova I Abisheva E Sabirova Z Beisenova Z. The development of individual self-efficiency among university students. Cypriot J Educ Sci. (2022) 17(2):615–24. 10.18844/cjes.v17i2.6857

[31] Skar JB Garnweidner-Holme LM Lukasse M Terragni L. Women’s experiences with using a smartphone app (the Pregnant+ app) to manage gestational diabetes mellitus in a randomised controlled trial. Midwifery. (2018) 58:102–8. 10.1016/j.midw.2017.12.021 29329023

[32] Abd-Elhakam EM Ramadan EA El-Houfey AA Abd El haliem Said S. Effect of an educational program based on health belief model on prevention of preterm birth among newly pregnant women. Int J Manage. (2020) 11(10):1029–45. 10.34218/IJM.11.10.2020.093

