# Supplementary material for: The effect of training intervention based on health belief model on self-care behaviors of women with gestational diabetes mellitus
Source: Front Glob Womens Health. 2025 Jan 17;5:1490754. doi: 10.3389/fgwh.2024.1490754 (PMC11782153; doi:10.3389/fgwh.2024.1490754)
Supplement: Supplementary file 1 [file Datasheet1.pdf]

Questionnaire for Assessing Self-Care Behaviors and Constructs of the Health Belief Model in Women with Gestational Diabetes Mellitus

---

Section A: Background Information

Health Record Number: ....

1. Age: .... years
2. Education Level:

- Illiterate □
- Below diploma □
- Diploma □
- University □

3. Occupation:

- Homemaker □
- Employed □
  Job Category: ....

4. Pre-pregnancy Weight: .... kilograms
5. Height: .... centimeters
6. Gestational Age: .... weeks
7. Parity (Number of Births): ....
8. Blood Glucose Level (Fasting): .... mg/dL
9. Blood Glucose Level (2 hours post-meal): .... mg/dL
10. Insulin Use (if applicable): Yes □ No □
11. Family History of Diabetes (First-degree relatives): Yes □ No □
12. History of Macrosomia (Large Infant at Birth): Yes □ No □

Section B: Knowledge Assessment

For each statement, choose one option: Yes □ No □ No Idea □

1. Gestational diabetes mellitus (GDM) occurs only during pregnancy and may resolve after delivery.
2. GDM increases the risk of complications for both the mother and the baby.
3. Women with GDM have a higher chance of developing Type 2 diabetes later in life.
4. Regular monitoring of blood glucose levels can prevent complications in women with GDM.

5. A balanced diet, including foods with a low glycemic index, is important for managing GDM.
6. Physical activity during pregnancy can help control blood glucose levels in women with GDM.
7. Blood glucose levels should be measured both fasting and two hours after meals in GDM.
8. Insulin therapy is sometimes necessary to manage GDM when diet and exercise are insufficient.
9. Untreated GDM can lead to complications such as preterm delivery and high birth weight.
10. Babies born to mothers with uncontrolled GDM are at risk of developing low blood sugar after birth.
11. Women with GDM should avoid sugary foods and drinks to help control blood sugar levels.
12. Eating several small meals throughout the day is better than having three large meals for GDM management.
13. Smoking can worsen the health outcomes for pregnant women with GDM and their babies.
14. Stress management can play a role in better controlling blood glucose levels during pregnancy.
15. GDM can be managed effectively through a combination of healthy eating, physical activity, and medical care.

Section C: Health Belief Model Constructs

Each item is scored on a 5-point Likert scale:
1 = Strongly Disagree
2 = Disagree
3 = Neutral
4 = Agree
5 = Strongly Agree

Perceived Sensitivity (8 items)

1. I am at risk of developing complications from GDM if I do not follow self-care behaviors.
2. Women with GDM are more likely to develop Type 2 diabetes in the future.

3. My lack of physical activity increases my risk of GDM complications.
4. If I do not monitor my blood glucose regularly, I could harm my baby.
5. GDM can cause serious complications if not managed properly.
6. My family history of diabetes increases my likelihood of having GDM-related complications.
7. Even mild changes in blood glucose can lead to serious issues during pregnancy.
8. Not controlling GDM may lead to future pregnancies being high-risk.

Perceived Severity (8 items)

1. GDM is a serious condition that can negatively impact my health and my baby's health.
2. Complications from GDM can lead to long-term health problems for me and my child.
3. Uncontrolled GDM can increase the likelihood of delivery complications.
4. GDM can increase the risk of my baby being admitted to the neonatal intensive care unit.
5. High blood glucose during pregnancy can lead to permanent health problems for me.
6. My baby could develop health issues if I do not manage GDM properly.
7. GDM can lead to serious complications such as preterm delivery or high birth weight.
8. GDM increases the likelihood of surgical delivery due to complications.

Perceived Benefits (10 items)

1. Monitoring my blood glucose levels regularly can help me avoid complications.
2. Following a healthy diet can help me control my blood sugar levels.
3. Engaging in regular physical activity will improve my overall pregnancy health.
4. Insulin therapy can effectively control high blood glucose levels during pregnancy.
5. Adhering to my healthcare provider's advice can reduce the risks associated with GDM.
6. Proper management of GDM will protect my baby's health.
7. Participating in educational sessions has improved my understanding of GDM.
8. A supportive family environment can help me better manage GDM.
9. Keeping a record of my self-care behaviors helps me stay consistent in managing GDM.
10. Learning about GDM motivates me to take care of my health.

Perceived Barriers (8 items)

1. Monitoring my blood glucose levels is difficult due to lack of time or access to equipment.
2. Preparing healthy meals for GDM is too costly or time-consuming.
3. Exercising regularly during pregnancy is uncomfortable or inconvenient.
4. Insulin injections are painful and hard to administer.
5. Family obligations make it challenging to focus on my self-care.
6. Lack of knowledge about GDM makes it hard to manage my condition.
7. Stress and emotional factors prevent me from following my self-care routine.
8. Cultural norms make it difficult to prioritize my dietary needs.

Perceived Self-Efficacy (12 items)

1. I feel confident in my ability to monitor my blood glucose levels daily.
2. I can follow a healthy diet even during busy days.
3. I can consistently engage in physical activities suitable for pregnancy.
4. I am capable of administering insulin injections as prescribed.
5. I am confident in discussing my condition and progress with healthcare providers.
6. I can manage stress effectively to keep my blood glucose levels under control.
7. I can recognize symptoms of high or low blood glucose.
8. I can document my self-care activities daily.
9. I can seek support from my family when needed.
10. I feel confident in attending all follow-up appointments.
11. I am confident I can avoid unhealthy food choices.
12. I can sustain these self-care behaviors throughout my pregnancy.

Act Directions (7 items)

1. I will monitor my blood glucose levels daily.
2. I will prepare and eat meals according to my dietary plan.

3. I will participate in physical activity regularly.
4. I will take my medications and insulin as prescribed.
5. I will document my blood glucose levels and other self-care activities.
6. I will attend all follow-up visits with my healthcare provider.
7. I will actively seek knowledge about managing GDM.

Section D: Self-Care Behaviors

(For each behavior, choose one option: Yes □ No □)

1. I have monitored my blood glucose levels daily in the past week.
2. I have eaten a healthy diet in the past week.
3. I have engaged in physical activity in the past week.
4. I have taken my prescribed medications or insulin regularly in the past week.
5. I have documented my blood glucose levels and self-care activities in the past week.
6. I have avoided sugary foods and drinks in the past week.
7. I have discussed my condition with healthcare providers in the past week.
8. I have checked my blood pressure regularly in the past week.
9. I have avoided smoking or exposure to second-hand smoke in the past week.
10. I have kept a record of my dietary intake in the past week.
11. I have managed stress effectively in the past week.
12. I have sought family support for my GDM management in the past week.
13. I have followed my healthcare provider’s recommendations for self-care.
14. I have reviewed my weight and pregnancy progress with my healthcare provider.
15. I have attended all scheduled prenatal visits.
