# Supplementary material for: The effect of training intervention based on health belief model on self-care behaviors of women with gestational diabetes mellitus
Source: Front Glob Womens Health. 2025 Jan 17;5:1490754. doi: 10.3389/fgwh.2024.1490754 (PMC11782153; doi:10.3389/fgwh.2024.1490754)
Supplement: Supplementary file 2 [file Datasheet2.pdf]

Overview of the Training Program

The training program was designed as a structured intervention to improve self-care behaviors in women with gestational diabetes mellitus (GDM). Grounded in the principles of the Health Belief Model (HBM), the program aimed to enhance participants' knowledge, confidence, and motivation to manage their condition effectively. It consisted of six sessions, each lasting 50-55 minutes, conducted three times a week over a two-week period. The sessions were led by a multidisciplinary team, including a Ph.D. expert in health education and promotion, a specialist in adult endocrinology, a nutritionist, and a gynecologist.

Session Content and Details

Session 1: Introduction to Gestational Diabetes Mellitus

This session provided a foundational understanding of GDM, focusing on:

- The causes and risk factors of GDM, such as obesity, family history of diabetes, and advanced maternal age.
- Potential complications for the mother, such as increased risk of type 2 diabetes, and for the fetus, including macrosomia and preterm birth.
- The importance of early and consistent management of blood glucose levels to reduce complications.
- An overview of the Health Belief Model (HBM) as a framework for self-care, emphasizing constructs like perceived severity, perceived barriers, and self-efficacy.

Session 2: Dietary Management

Participants received comprehensive education on dietary changes to manage GDM, including:

- Principles of a balanced diet tailored to GDM, highlighting the selection of low-glycemic-index foods and limiting high-sugar snacks.
- Strategies for portion control and balancing macronutrients (carbohydrates, proteins, and fats) in daily meals.
- Practical guidance on meal planning, with examples of GDM-friendly recipes.
- The role of vitamins and minerals in supporting fetal development and maternal health.

This session included a demonstration of meal preparation and a discussion on overcoming common dietary challenges, such as food cravings and lack of time for meal planning.

Session 3: Physical Activity

Participants were educated on the role of physical activity in managing GDM, with a focus on:

- Types of safe exercises for pregnancy, including walking, prenatal yoga, and light aerobic activities.

- The benefits of regular physical activity for regulating blood glucose levels and improving overall well-being.
- Guidelines for the frequency, intensity, and duration of exercises appropriate for women with GDM.
- Practical tips for integrating physical activity into daily routines, especially for those with limited time or physical discomfort.

Session 4: Monitoring Blood Glucose

This session provided hands-on training on monitoring blood glucose levels, including:

- A step-by-step demonstration of using glucometers for fasting and postprandial blood glucose measurements.
- Guidance on interpreting glucose readings and identifying abnormal patterns.
- The importance of maintaining a consistent monitoring schedule and recording results for review.
- Addressing barriers to blood glucose monitoring, such as fear of needles or lack of confidence in using devices.

Participants practiced using glucometers during the session, with facilitators providing individualized feedback to ensure competence.

Session 5: Medication, Insulin Management, and Lifestyle Modifications

This session covered the pharmacological aspects of GDM management and lifestyle adjustments:

- Proper techniques for insulin injection, including preparation, administration, and storage of insulin.
- Importance of adherence to prescribed oral medications and insulin therapy.
- Addressing misconceptions or fears related to insulin use, such as dependency or adverse effects.
- Education on the harmful effects of smoking during pregnancy and strategies for cessation.
- Stress management techniques, including relaxation exercises, to support mental well-being.

Session 6: Family and Community Support

The final session emphasized the importance of a supportive environment in sustaining self-care behaviors:

- Family members, including husbands, healthcare staff, and diabetes specialists, were invited to participate.
- Strategies to foster a supportive family and community environment for women with GDM.
- A recap of the program’s key messages and encouragement to continue practicing self-care behaviors post-training.

Participants were provided with educational materials, including a training booklet summarizing the session content, practical tips for self-care, and GDM-friendly recipes. A CD with instructional videos on blood glucose monitoring, meal preparation, and exercise routines was also distributed.

Interactive Methods

The program utilized interactive learning methods to actively engage participants. These included:

- Group discussions and role-playing to simulate real-life scenarios and address participants' concerns.
- Practical demonstrations, such as using glucometers, planning meals, and insulin administration, to build confidence and competence.
- Question-and-answer sessions to clarify doubts and reinforce understanding.

Self-Documentation

Participants were asked to document their daily self-care activities in structured forms, including:

- Fasting and postprandial blood glucose readings.
- Details of meals consumed and physical activities performed.
- Insulin dosages and adherence to medications.
- Challenges faced and solutions implemented.

This self-documentation was reviewed by facilitators during follow-up interactions to provide personalized feedback and guidance.

Follow-Up Strategy

A six-week follow-up period was implemented to sustain behavioral changes and reinforce learning. Weekly telephone calls and in-person reminders were conducted to:

- Address participants' challenges and provide additional support.
- Monitor adherence to self-care behaviors and identify areas for improvement.
- Motivate participants to continue practicing healthy habits.

Program Objectives

The primary objectives of the training program were:

- To improve participants' knowledge about GDM and its management.
- To enhance self-efficacy and confidence in adopting self-care behaviors.
- To reduce perceived barriers and increase adherence to dietary, physical activity, and medication recommendations.
- To achieve sustainable improvements in blood glucose control and overall clinical outcomes.
